# Ecotoxicity Study of Additives Composed of Zinc and Boron

**DOI:** 10.3390/toxics10120795

**Published:** 2022-12-17

**Authors:** Šárka Petrová, Petr Soudek

**Affiliations:** Laboratory of Plant Biotechnologies, Institute of Experimental Botany of the Czech Academy of Sciences, Rozvojová 263, 165 02 Prague, Czech Republic

**Keywords:** zinc borate, duckweed, lettuce, growth inhibition

## Abstract

The high use of additives containing zinc borate and their limited solubility in water both lead to its persistence and accumulation in biological systems. On the other hand, soluble forms of boron are easily available to plant roots and are taken up by plants. There are no ecotoxicological data available for zinc borate, the industrial utilization of which is widespread. Therefore, the potential toxicity of zinc borate and its dissociated compounds was evaluated. Based on two different ecotoxicology tests, their effect on plant growth was studied. Firstly, the impact on *Lemna minor* growth was investigated, including the effect on pigment content. Secondly, the inhibition of the root growth of higher plant species *Sinapis alba* (mustard), *Lactuca sativa* (lettuce) and *Trifolium pretense* (clover) was measured. The growth inhibition test on *L. minor* was more complex and sensitive compared to the plant seed germination test. Already low concentrations (10 mg/L) of ZnO, B_2_O_3_ and Zn_3_BO_6_ led to a decrease in frond growth and to an inhibition of the conversion of chlorophyll a to chlorophyll b. These results suggested that the stress caused by these additives caused damage to the photosynthetic apparatus. The highest inhibition of frond growth was detected in fronds treated with B_2_O_3_ (92–100%). In ZnO and Zn_3_BO_6_, the inhibition of frond growth was between 38 and 77%, with Zn_3_BO_6_ being slightly more toxic. In the seed germination test, the most sensitive species was lettuce, the growth of which was inhibited by 57, 83 and 53% in ZnO, B_2_O_3_ and Zn_3_BO_6_ treatments, respectively. However, the inhibitory effect on each plant was different. In lettuce and clover, the seed germination and root elongation decreased with increasing element concentrations. In contrast, in mustard, low concentrations of ZnO and Zn_3_BO_6_ supported the growth of roots. For that reason, more complex tests are essential to evaluate the additive toxicity in the environment.

## 1. Introduction

Boron compounds (borates, boric acid) have been extensively used as flame retardants, smoke suppressants, afterglow suppressants and antitracking agents since 1970 [1]. Zinc borate, as an inorganic flame retardant with a low cost, forms a glassy substance on the burning material surface, accompanied by a release of water. It is typically composed of 45% zinc oxide (ZnO) and 34% boric anhydride (B_2_O_3_), with about 20% water content. ZnO is widely utilized for its properties (high refractive index, high thermal conductivity, binding, antibacterial and UV-protection properties) as an additive that enhances the flame retardancy of phenolic foams, as a halogen-free flame retardant system consisting of ammonium polyphosphate, as a blowing agent for pentaerythritol and as a carbonific agent [2].

The high usage of boron additives and their limited water solubility leads to their persistence and accumulation in biological systems. Few studies have examined the environmental impacts of flame retardants on life cycle [3,4]. Screening studies dealt with data from existing databases and model programs [5,6,7,8] that estimated chemicals' acute and chronic toxicity [9]. It was shown that uncertainty in persistence data contributes most to the uncertainty in the bioaccumulation and toxicity of chemicals [10].

Some important questions regarding the environmental impact of zinc borate remain unresolved, despite its widespread and growing industrial use. Zinc borate is relatively immobile in the environment due to low water solubility and low vapor pressure. Transport is more likely to occur in water at low pH, where Zn_3_BO_6_ dissociates into zinc and borate ions.

Soluble forms of boron (at pH 5.5–7.5 undissociated H_3_BO_3_) are easily available to plants by root uptake through passive diffusion across lipid layers, by protein channels and by the energy-dependent high-affinity transport system [11]. Boron is an essential element involved in cell-wall bindings; the conversion of sugars to starch, lignin and flavonoid synthesis; and the metabolism of auxins, nitrogen compounds, phenols and nucleic acids [12,13]. On the other hand, H_3_BO_3_ was recommended as a reference substance in several international standard soil ecotoxicity test methods [14]. H_3_BO_3_ deficiency affects ascorbate metabolism [15]. Excessive amounts may be toxic to aquatic organisms, as well as plant species [16,17]. For example, the frond production of *S. polyrrhiza* was significantly reduced when a boron concentration of 3.55 mg/L was applied to the medium [18] or when *L. minor* frond production was reduced at concentrations above 16 mg/L.

In plants, zinc is essential for the protection and maintenance of the structural stability of cell membranes, and it plays an important role in biomass production, chlorophyll production, and germination [19]. The positive effects of ZnO nanoparticles on plant growth have been widely described in the literature [20]. On the other hand, high zinc levels (above 10 mg/L) dissolved in water caused oxidative stress and affected the photosynthetic performance of *Lemna* spp., while they did not affect frond development [21]. ZnO nanoparticles in alkaline water are not toxic to *L. minor* due to lower dissociation [22]. In accordance, it has been shown that ZnO toxicity is mainly related to soluble zinc rather than to particle size [23]. That means that both bulk ZnO and ZnO nanoparticles may be toxic when dissolved [24,25].

In general, toxicological studies on fire retardants focus mainly on the toxic effects of substances or their combustion products on human health [26]. While only a few studies have examined the environmental impact of such additives in life-cycle assessment studies [3,4], there are no data available for the ecotoxicity of zinc borate.

Standardized toxicity tests focus on freshwater aquatic plants of the genus *Lemna* (tested substance in the water) or on seedling emergence and the early growth of higher plants (tested substance in the soil) [27,28]. As mentioned above, zinc concentrations in water above 10 mg/L caused oxidative stress and affected the photosynthesis of *Lemna* spp., while its frond development stayed unaffected [21]. On the other hand, the frond production of *L. minor* was inhibited by soluble boron in a concentration above 16 mg/L [18]. The reduction in chlorophyll a, chlorophyll b and carotenoid contents in mustard plants was also observed in the presence of high boron concentrations. However, data on the ecotoxicity of zinc borate are missing. Gathered information on its ecotoxicity should serve as a signal for ecosystem services analyses. The aim of this study was to determine the possible toxicity of zinc borate and its dissociated products in plants. Two different tests were applied to determine the influence of zinc borate and its dissociated compounds on plant growth.

## 2. Materials and Methods

The chemicals for the cultivation solutions were of analytical grade and obtained from Penta Chemicals Unlimited (CR). Tested substances were obtained from Sigma-Aldrich (USA): ZnO 100 nm particles, CAS: 1314-13-2, catalog No: 544906; B_2_O_3_, CAS: 1303-86-2, catalog No: 15678; Zn_3_BO_6_, CAS: 10361-94-1, catalog No: 14470.

### 2.1. Lemna Minor Growth Inhibition Test

The objective of this test is to quantify substance-related effects on vegetative growth based on the assessments of frond number, wherein the assessments are expressed as the growth inhibition of duckweed (*L. minor*) [29]. Plants of *L. minor* (Federal Environmental Research, Berlin, Germany) were cultivated under sterile conditions in 250 mL vessels with 100 mL of Steinberg solution (Table S1) in a cultivation room (25 °C, average irradiation 72 µmol/m^2^ s^1^ at the plants’ surfaces, with horizontal differences in irradiation less than 20%). During the experiment, 5 replicates for control and 3 replicates for each chemical (ZnO 100 nm particles, B_2_O_3_, Zn_3_BO_6_) at concentrations of 10, 50, 100, 250, 500 and 1000 mg/L were applied. Healthy frond colonies of duckweed that were dark green and consisted of two or three identical leaves were selected for the experiments. The number of fronds and their areas were calculated at the start of the experiments (12 fronds for each beaker) and then, after 4 and 7 days of treatment, the software NIS Elements Ar 4.11 (Nikon) was used. The relative growth rate was calculated for both frond number and frond area. The percentual inhibition of the growth rate for each tested concentration was calculated from the average growth rate of the control and of the treated group.

The average specific growth rate (µ) for a 7-day period was calculated as the logarithmic increase in the growth variables—frond numbers and total frond area—using the formula below, for each replicate of control and treatments:

$$\mu =\frac{lnN_{n}-\ln N_{0}}{t_{n}}$$

where *N_n_* is the final number of leaves (or the final area), *N*_0_ is the initial number of leaves (or the initial area) and *t_n_* is the duration of the test.

Pigment contents were measured in all fronds from each treatment (sample weight ranged from 10 to 30 mg of fresh weight; balances KERN ALT220-5DAM, Balingen, Germany). Pigments were extracted overnight in the dark at 4 °C by placing fronds into 10 mL pure methanol (Reag. Ph. Eur. For HPLC- gradient grade, VWR°Chemicals, CR). After centrifugation, the extracts were determined spectrophotometrically (Infinite 200 Microplate Reader, Tecan, Grödig, Austria) at the following wavelengths: 665.2, 652.4 and 470 nm [30]. Concentrations were calculated according to the following formula:

$$c_{a}=16.72\times A_{665.2}-9.16\times A_{652.4}$$

$$c_{b}=34.09\times A_{652.4}-15.28\times A_{665.2}$$

$$c_{x+c}=\frac{\left(1000\times A_{470}-1.63\times c_{a}-104.96\times c_{b}\right)}{221}$$

where $c_{a}$ is chlorophyll a, $c_{b}$ is chlorophyll b, $c_{x+c}$ means carotenoids, and $A_{652.4}$, $A_{665.2}$ and $A_{470}$ are absorbances at 652.4 nm, 665.2 nm and 470 nm, respectively.

The percentual inhibition (I) of chlorophyll synthesis was calculated according to the following formula:

$$I=\frac{c_{c}-c_{s}}{c_{c}}*100$$

where $c_{c}$ is the concentration of the pigment in the control plants, and $c_{s}$ is the concentration of the pigment in the plants treated with ZnO, B_2_O_3_ or Zn_3_BO_6_, respectively.

### 2.2. Acute Toxicity Test

The effect of contaminants on the seed germination and root growth in the early stages of development was studied [31,32,33]. The toxicity of chemicals (ZnO 100 nm particles, B_2_O_3_, Zn_3_BO_6_), diluted in nutrient solutions at concentrations of 10, 50, 100, 250, 500 and 1000 mg/L, was tested on seeds of white mustard (*Sinapis alba*), lettuce (*Lactuca sativa*) and clover (*Trifolium pratense*). Plant species were selected from the OECD 208 list of test plant species. Seventeen undamaged and plump seeds with almost identical size were placed uniformly on the surface of the filter paper at the bottom of each dish, which contained 5 mL of solution. All dishes were incubated in the dark in a temperature-constant incubation room. All experiments were performed in four replicates. After 72 h, root length was measured and percentual inhibition (*I_r_*) was calculated according to the following formula:

$$I_{r}=\frac{\mu_{c}-\mu_{t}}{\mu_{c}}*100$$

where $\mu_{c}$ is the growth rate of the reference sample, and $\mu_{t}$ is the growth rate of tested sample (Table S2).

### 2.3. Data Analysis

The relative responses were expressed as mean ± standard deviation. A two-way ANOVA test with Dunnett’s multiple comparisons (Statistica 12, Stat soft., Inc., Tulsa, OK, USA) was used to test for significant differences among the parameters.

## 3. Results and Discussion

The potential toxicity of chemicals was estimated based on two different tests. Firstly, a *Lemna* sp. growth inhibition test was employed, as it is recommended by the OECD [28] and because it is the most standardized plant bioassay for the assessment of the impact of contaminants on an aquatic environment [34]. In addition, the seed germination test determined the toxicity of chemicals to the root growth of higher plants.

### 3.1. Lemna Minor Growth Inhibition Test

**Figure 1:**
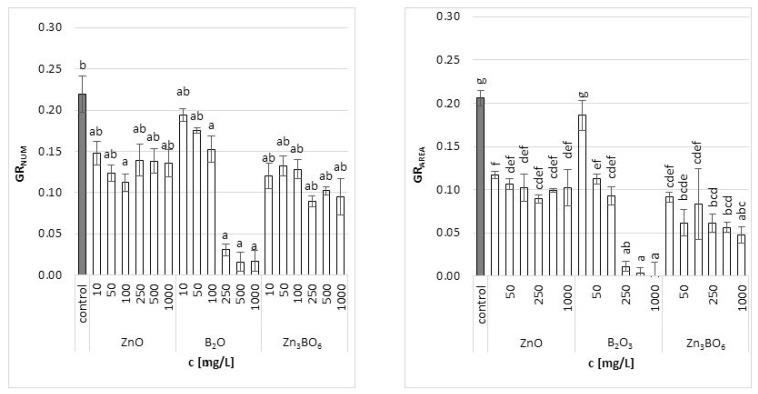
The relative growth rate of *L. minor* plants (based on counted frond number GR_NUM_ and frond area GR_AREA_) after 7 days of growth in the solutions supplemented with ZnO, B_2_O_3_ or Zn_3_BO_6_, respectively, at concentrations of 10, 50, 100, 250, 500 and 1000 mg/L. Control plants were grown in Steinberg solution; standard deviation is represented as ± S.D. (*n* = 3), and two-way ANOVA test with Dunnett’s multiple comparisons was applied. The average growth rate was calculated as the slope of the logarithmic growth curve.

Both growth parameters (number of fronds and their area) determined in this study yielded similar trends of specific growth rates. There was a significant decrease in the relative growth rate of *L. minor* with increasing concentration of tested chemicals (Figure 1). The results, however, showed differences between concentrations and substances. The most pronounced effect was observed in B_2_O_3_ treatment. When B_2_O_3_ was applied, both the number of fronds, as well as their area, decreased steeply with increasing concentration. While the effect of the lowest concentration used (10 mg/L) was comparable to control, the concentrations above 250 mg/L were almost lethal to the plant. Other two treatments (ZnO, Zn_3_BO_6_) decreased the growth rate at the lowest concentration, but the decrease was very similar for all concentrations applied. In case of ZnO, data based on the number of fronds showed a decrease in growth with increasing concentration up to 100 mg/L; however, the effect of the higher concentrations was similar to the effect of the 10 mg/L concentration. In case of Zn_3_BO_6_, its higher concentrations caused a more pronounced decrease in the number of fronds; however, the decrease was still far from lethal. The effect of the increasing concentration was more significantly visible on the results based on the frond area. The higher the concentration applied, the superior frond area inhibition was caused (Figure 1).

Calculated values for the growth rate inhibition (based on frond number or frond area) showed that the toxic effect of additives increased with increasing concentrations (Table 1). The highest growth inhibition was detected in fronds treated with B_2_O_3_ (92% or 100% for frond number and area, respectively). The inhibition of frond growth in the case of ZnO was 38% (by number of fronds) and 50% (by frond area), while, in the case of Zn_3_BO_6_, it was almost 57% and 77%, respectively. Similar results were published for *S. polyrrhiza* plants, in which increased boron concentrations (above 22.4 mg/L) significantly increased abnormal frond growth [18]. Another study showed that increasing boron concentrations caused a decrease in the growth rate of *L. gibba* plants at a boron level of 25 mg/L [35]. Matching with our results, no toxicity symptoms were observed in plants exposed to boron concentrations up to 10 mg/L [35,36,37]. On the other hand, zinc concentrations ranging between 4 and 50 mg/L inhibited the growth of *L. gibba* by 50–79% [38], which was slightly higher than the growth inhibition observed in our study. The excess of zinc available to plants was also reported to induce oxidative stress and antioxidant response in plants, led to a decrease in root elongation and the chlorosis of leaves [39,40,41] and affected the photosynthetic performance of *Lemna* spp. [21].

A comparison of the results showed that the calculation based on frond area was more reliable (Table 1). Due to the fact that the area of fronds is a continuous variable, while the number of fronds increases discontinuously, the frond area seems to be a more stable parameter to measure the growth rate, while the frond number remains important as a basic parameter that is always accessible [42,43]. Therefore, the pigment values were further related to the frond area.

**Figure 2:**
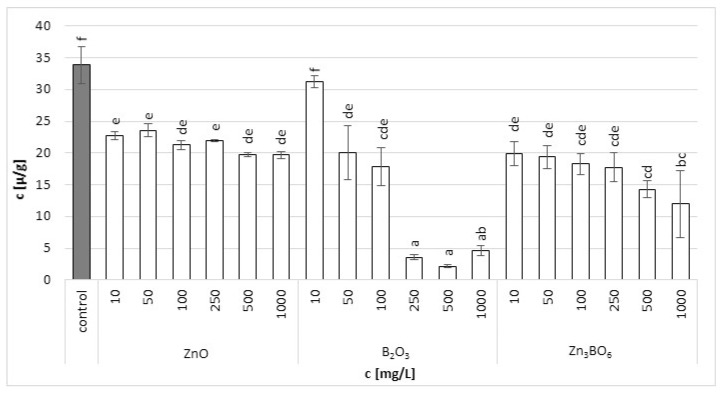
Chlorophyll a content in *L. minor* plants after 7 days of growth in the solutions supplemented with ZnO, B_2_O_3_ or Zn_3_BO_6_ at concentrations of 10, 50, 100, 250, 500 and 1000 mg/L. Control plants grew in Steinberg solution; standard deviation is represented as ± S.D. (*n* = 3), and two-way ANOVA test with Dunnett’s multiple comparisons was applied.

**Figure 3:**
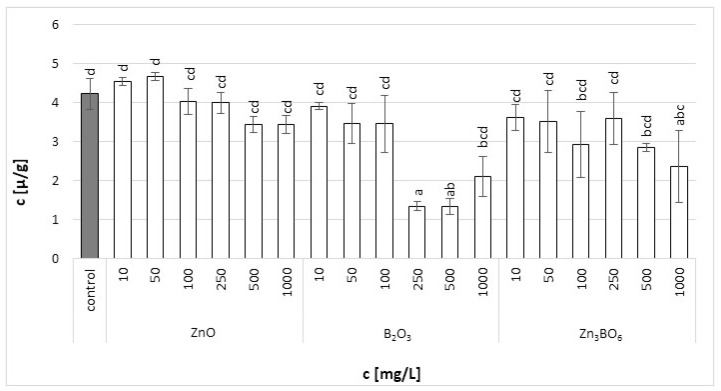
Chlorophyll b content in *L. minor* plants after 7 days of growth in the solutions supplemented with ZnO, B_2_O_3_ or Zn_3_BO_6_, respectively, at concentrations of 10, 50, 100, 250, 500 and 1000 mg/L. Control plants grew in Steinberg solution; standard deviation is represented as ± S.D. (*n* = 3), and two-way ANOVA test with Dunnett’s multiple comparisons was applied.

Chlorophyll a in the fronds of *L. minor* decreased with increasing additive concentrations (Figure 2). The fronds treated with B_2_O_3_ at concentrations above 250 mg/L were seriously damaged. In the case of ZnO and Zn_3_BO_6_ treatment, the toxic effect was not visible, but the increasing concentrations decreased the amount of chlorophyll a in the fronds. Similar results were obtained for chlorophyll b. At lower ZnO concentrations, the chlorophyll b content increased with ZnO concentration, probably because chlorophyll a was converted to chlorophyll b (Figure 3). However, at concentrations above 100 mg/L, the chlorophyll b content decreased with increasing ZnO concentration in the medium. Decreased chlorophyll a/b ratio values (Figure S1) were independent of ZnO or Zn_3_BO_6_ concentration in medium. This could be due to the lower solubility of the tested substances. At concentrations above 10 mg/L, ZnO and Zn_3_BO_6_ decreased the chlorophyll a/b ratio by 1.35–1.65-fold and 1.65–2.7-fold, respectively. The most toxic effect was again observed on fronds grown in the medium supplemented with B_2_O_3_. At concentrations of 500 and 1000 mg/L, B_2_O_3_ decreased the chlorophyll a/b ratio by 4.8-fold and 4-fold, respectively. The conversion of chlorophyll a to chlorophyll b indicated stress and damage to the photosynthetic apparatus in *L. minor* fronds [44]. Our results show that the presence of zinc ions reduces the toxic effect of boron. The concentration of Zn_3_BO_6_ above 250 mg/L decreased the chlorophyll a and b content, but the fronds were not as seriously damaged as the ones grown in the medium supplemented with B_2_O_3_. The inhibition data showed that the most toxic compound was B_2_O_3_, whereby the content of chlorophyll a and b decreased up to 93% and 69%, respectively (Table 2). The decrease in chlorophyll a and b contents caused by Zn_3_BO_6_ reached 65% and 44%, respectively. Only in the case of ZnO did the decrease in chlorophyll contents not reach 50%. The decrease was 42% and 19% for chlorophyll a and chlorophyll b, respectively.

**Figure 4:**
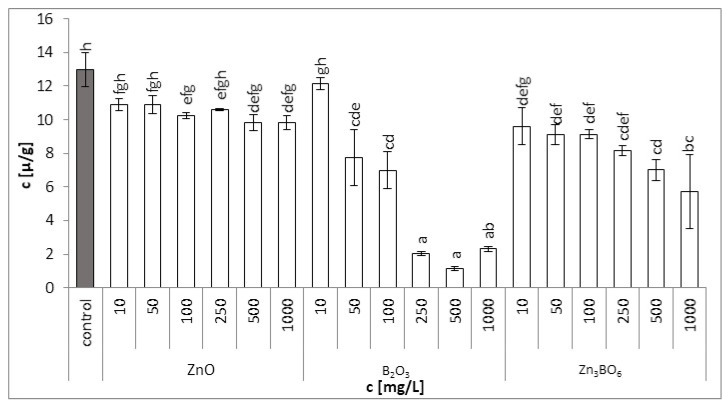
Total carotenoid contents in *L. minor* plants after 7 days of growth in the solutions supplemented with ZnO, B_2_O_3_ or Zn_3_BO_6_, respectively, at concentrations of 10, 50, 100, 250, 500 and 1000 mg/L. Control plants grew in Steinberg solution; standard deviation is represented as ± S.D. (*n* = 3), and two-way ANOVA test with Dunnett’s multiple comparisons was applied.

Similarly, total carotenoid content in plants treated with B_2_O_3_ was affected more significantly than with the ZnO or Zn_3_BO_6_ treatments. At concentrations above 250 mg/L, total carotenoid content decreased 10 times (Figure 4). Only the lowest concentration (10 mg/L of B_2_O_3_) had no effect on total carotenoid content, with the value comparable to control. Total carotenoid content (Table 2) decreased by 24%, 55% and 91% for treatments with ZnO, Zn_3_BO_6_ and B_2_O_3_, respectively.

Generally, a gradual decrease in chlorophyll and carotenoid contents was observed with increasing concentrations of ZnO, B_2_O_3_ or Zn_3_BO_6_. Our findings were in agreement with other studies that reported changes in photosynthetic pigment and carotenoid contents and their relative ratios caused by excessive boron concentrations [45,46]. Photosynthesis is one of the main metabolic processes disturbed by an excess of boron [47]. The slower development of the photosynthetic apparatus in young fronds and the lower yield of photosynthesis caused by boron was reported [48]. The excess of boron can result in damage to the photosynthetic apparatus and in a negative effect on the process of the photosynthetic electron transport ratio in duckweed tissues [49]. Similarly, earlier studies showed that stress caused by zinc ions inhibited the synthesis of chlorophyll in plant leaves [50]. It was reported that 10 mg/L of ZnO nanoparticles caused a decrease in the maximal quantum yield of photosystem II efficiency values compared to the untreated control [22]. The zinc toxicity mechanism was reported in the damage to the structure of the thylakoid membrane, which reduces either chlorophyll content or the substitution of the central manganese ion of chlorophyll [51]. On the other hand, zinc application significantly reduced boron toxicity due to the inhibition of its uptake in a rice–wheat cropping system [52]. Our results also indicated that zinc application appeared to create a protective mechanism in the root cell environment against the excessive uptake of boron in wheat plants.

### 3.2. Acute Toxicity

The primary purpose of the seed germination study was the determination of acute toxicity. The performed test with seeds of white mustard (*Sinapis alba*), lettuce (*Lactuca sativa*) and clover (*Trifolium pratense*) was much less sensitive than the test on duckweed, but it extended the testing to higher plants. The inhibition of root growth was measured. The most sensitive species was lettuce, with the highest inhibition of root growth, by 56.4, 82.8 and 53.2% for ZnO, B_2_O_3_ and Zn_3_BO_6_, respectively (Table 3, Table 4 and Table 5). Clover seed germination was comparable with the control treatment up to concentrations of 100 mg/L. With increasing additive concentration, the inhibition of root growth increased up to 44% for ZnO and Zn_3_BO_6_ treatments and up to 67% for B_2_O_3_ treatment. Low concentrations of ZnO and Zn_3_BO_6_ did not show statistical differences in calculated inhibitions for clover, and inhibition values were highly variable but the interval was comparable with control treatment. The high variability in the ZnO toxicity effect was in accordance with Lin et al., 2007 [53]. Mustard seeds were the least sensitive and their root growth was inhibited only in the case of B_2_O_3_ (Table 4) at concentrations of 500 mg/L (by 11%) and 1000 mg/L (by 36%). On the contrary, ZnO and Zn_3_BO_6_ treatments supported the seed germination and root growth of mustard. At a concentration of 100 mg/L ZnO, root growth increased by almost 97%. Zn_3_BO_6_ increased root growth by more than 100% at all concentrations applied except for the highest (Table 5). Zinc application might led to the induction of a protective mechanism against the excessive uptake of boron. Our results are in accordance with the published study on wheat plants [54]. It was also reported that zinc treatments reduced the inhibitory effect of boron on the growth of tomato [55]. The combination of boron and zinc significantly increased rice growth and yield, as well as chlorophyll content and boron assimilation [56]. These elements were antagonists; therefore, zinc application has been recommended for alleviating boron toxicity on boron-rich soils [57,58]. The root growth of higher plants was affected the most with B_2_O_3_, which is comparable to its effect on duckweed. The root growth inhibition could be caused by the reduction in the meristem size due to the reduction in cell division [59,60] or by the disruption of cytoplasmic metabolism, resulting from boron toxicity [61]. It was also reported that the high doses of boron fertilizer increased enzymatic antioxidant activity in lettuce in order to decrease the stress damage [62]. A previously published study identified the root tip as a site of boron toxicity because an inhibition of root growth occurred if the excess of boron was applied to the root tip region but not if the excess of boron was applied to the mature sections of the root [63].

## 4. Conclusions

The potential toxicity of zinc and boron additives was estimated based on two different ecotoxicology tests. First, the quantified semichronic toxicity on the vegetative growth of *L. minor* was tested. The second test extended the testing to higher plants. The additives' toxicities decreased in both tests in the following order: B_2_O_3_, Zn_3_BO_6_, ZnO. It was shown that the inhibitory effect of boron can be reduced by the presence of zinc ions. The growth inhibition test on *L. minor* was more complex and sensitive when compared to the plant seed germination test on higher plants. The highest concentration of B_2_O_3_ inhibited *L. minor* frond growth up to 100%. Even the low concentrations of tested compounds led to a decrease in frond growth and to a conversion of chlorophyll a to chlorophyll b, indicating stress and damage to the photosynthetic apparatus. The sensitivity of higher plant seeds was not as pronounced, but the dependence on plant species was very high. Seed sensitivity decreased in the order lettuce—clover—mustard. In this context, it is necessary to compare several plant species to determine whether the substances tested pose a potential risk to the environment. It can be concluded that boron toxicity can be classified as a risk to plants, and therefore its concentration in the environment should be monitored, and further life cycle assessment studies should be carried out.

## Figures and Tables

**Table 1:** The inhibition of growth of *L. minor* plants (based on counted frond number and frond area) after 7 days of growth in the solutions supplemented with ZnO, B_2_O_3_ or Zn_3_BO_6_ at concentrations of 10, 50, 100, 250, 500 and 1000 mg/L. Control plants grew in Steinberg solution; standard deviation was represented as ± S.D. (*n* = 3), and two-way ANOVA test with Dunnett’s multiple comparisons was applied.

Additive	ZnO				B_2_O_3_				Zn_3_BO_6_			
c [mg/L]	I _NUM_ [%]		I _AREA_ [%]		I _NUM_ [%]		I _AREA_ [%]		I _NUM_ [%]		I _AREA_ [%]	
10	32.5	± 6.57 ^a,b,c,d^	43.1	± 2.02 ^b^	11.5	± 3.68 ^a^	9.79	± 8.20 ^a^	45.2	± 7.15 ^c,d,e,f^	55.6	± 2.82 ^b,c,d,e^
50	43.7	± 4.51 ^c,d,e,f^	48.3	± 3.28 ^b,c,d^	20.1	± 1.31 ^a,b^	45.4	± 2.80 ^b,c^	39.7	± 5.51 ^b,c,d,e,f^	70.0	± 7.41 ^c,d,e,f^
100	48.8	± 4.85 ^c,d,e,f^	50.2	± 7.57 ^b,c,d^	30.4	± 7.40 ^a,b,c^	54.8	± 5.06 ^b,c,d,e^	41.4	± 5.13 ^b,c,d,e,f^	59.4	± 19.9 ^b,c,d,e^
250	36.4	± 8.78 ^b,c,d,e^	56.6	± 2.37 ^b,c,d,e^	85.8	± 3.24 ^g^	94.5	± 2.86 ^f,g,h^	59.0	± 2.99 ^f^	71.6	± 5.00 ^c,d,e,f^
500	37.0	± 6.70 ^b,c,d,e,f^	51.8	± 1.08 ^b,c,d,e^	92.7	± 5.22 ^g^	98.5	± 3.31 ^g,h^	53.4	± 2.12 ^d,e,f^	72.6	± 2.72 ^d,e,f,g^
1000	38.2	± 7.41 ^b,c,d,e,f^	50.5	± 10.2 ^b,c,d^	92.1	± 5.60 ^g^	101	± 8.55 ^h^	56.7	± 10.1 ^e,f^	76.8	± 4.36 ^e,f,g,h^

**Table 2:** The change in chlorophyll a and b content (calculated as a percentual inhibition of chlorophyll synthesis compared to control) in *L. minor* plants after 7 days of growth in the solutions supplemented with ZnO, B_2_O_3_ or Zn_3_BO_6_, respectively, at concentrations of 10, 50, 100, 250, 500 and 1000 mg/L. Control plants grew in Steinberg solution; standard deviation is represented as ± S.D. (*n* = 3), and two-way ANOVA test with Dunnett’s multiple comparisons was applied.

Additive	ZnO						B_2_O_3_						Zn_3_BO_6_					
c [mg/L]	I _CHL a_ [%]		I _CHL b_ [%]		I _CAROT_ [%]		I _CHL a_ [%]		I _CHL b_ [%]		I _CAROT_ [%]		I _CHL a_ [%]		I _CHL b_ [%]		I _CAROT_ [%]	
10	32.8	± 1.88 ^b^	−7.50	± 2.43 ^a^	16.2	± 2.68 ^a,b^	7.58	± 2.79 ^A^	7.35	± 1.96 ^A,B^	6.49	± 2.93 ^A^	41.2	± 5.39 ^β,γ^	14.4	± 7.72 ^α,β^	26.0	± 8.54 ^α,β,γ,δ^
50	30.4	± 2.95 ^b^	−10.7	± 7.98 ^a^	16.1	± 4.27 ^a,b^	40.7	± 12.4 ^B,C^	18.1	± 12.0 ^A,B^	40.2	± 12.9 ^C,D,E^	42.7	± 5.33 ^β,γ^	16.7	± 18.9 ^α,β^	29.9	± 4.48 ^β,γ,δ^
100	37.1	± 2.09 ^b^	4.40	± 4.95 ^a,b^	21.1	± 1.45 ^a,b,c^	47.4	± 8.83 ^B,C,D^	18.1	± 17.2 ^A,B^	46.1	± 8.31 ^D,E^	46.0	± 4.91 ^β,γ,δ^	30.8	± 20.1 ^α,β,γ^	29.6	± 2.06 ^β,γ,δ^
250	35.2	± 0.41 ^b^	5.33	± 6.39 ^a,b^	18.5	± 0.42 ^a,b,c^	89.4	± 1.06 ^F^	68.1	± 2.71 ^D^	84.5	± 0.88 ^G^	47.5	± 6.84 ^β,γ,δ^	14.9	± 15.8 ^α,β^	37.3	± 2.29 ^β,γ,δ,ε^
500	41.6	± 1.09 ^b,c^	15.2	± 4.95 ^a,b^	24.3	± 3.84 ^a,b,c,d^	93.7	± 0.71 ^F^	68.5	± 4.99 ^C,D^	91.2	± 2.93 ^G^	57.7	± 4.04 ^γ,δ^	32.3	± 2.35 ^α,β,γ^	46.0	± 4.75 ^δ,ε^
1000	41.8	± 1.44 ^b,c^	18.6	± 5.33 ^a,b^	24.3	± 3.36 ^a,b,c,d^	86.4	± 2.49 ^E,F^	50.0	± 12.2 ^A,B,C^	82.2	± 1.01 ^F,G^	64.6	± 15.4 ^δ,ε^	44.0	± 22.0 ^β,γ,δ^	55.9	± 17.0 ^ε,ζ^

**Table 3:** The inhibition of root elongation content (calculated as a percentual inhibition of root growth compared to control) of white mustard (*Sinapis alba*), lettuce (*Lactuca sativa*) and clover (*Trifolium pratense*) after 3 days of seed germination in a solution supplemented with ZnO at concentrations of 10, 50, 100, 250, 500 and 1000 mg/L. Standard deviation is represented as ± S.D. (*n* = 3), and two-way ANOVA test with Dunnett’s multiple comparisons was applied.

	I [%]					
c [mg/L]	*S. alba*		*L. sativa*		*T. pratense*	
10	−52.6	± 3.83 ^a^	12.8	± 4.00 ^C^	−0.317	± 4.36 ^α^
50	−71.4	± 4.26 ^c^	39.4	± 1.66 ^A^	−2.38	± 4.26 ^α^
100	−96.5	± 5.33 ^b^	34.7	± 2.73 ^A^	21.1	± 8.36 ^β^
500	−56.2	± 4.53 ^a^	51.0	± 1.66 ^B^	44.4	± 6.29 ^γ^
1000	−20.1	± 1.28 ^d^	56.4	± 3.31 ^B^	35.0	± 6.42 ^β,γ^

**Table 4:** The inhibition of root elongation (calculated as a percentual inhibition of root growth compared to control) of white mustard (*Sinapis alba*), lettuce (*Lactuca sativa*) and clover (*Trifolium pratense*) after 3 days of seed germination in a solution supplemented with B_2_O_3_ at concentrations of 10, 50, 100, 250, 500 and 1000 mg/L. Standard deviation is represented as ± S.D. (*n* = 3), and two-way ANOVA test with Dunnett’s multiple comparisons was applied.

	I [%]					
c [mg/L]	*S. alba*		*L. sativa*		*T. pratense*	
10	−55.9	± 8.70 ^a^	17.9	± 2.19 ^A^	1.27	± 3.44 ^α^
50	−50.3	± 3.76 ^a^	18.4	± 2.73 ^A^	−2.54	± 1.00 ^α^
100	−53.2	± 1.30 ^a^	14.8	± 2.82 ^A^	−13.9	± 2.35 ^β^
500	10.6	± 2.82 ^b^	39.8	± 6.26 ^B^	55.5	± 1.00 ^γ^
1000	36.3	± 4.10 ^c^	82.8	± 4.07 ^C^	66.9	± 2.62 ^δ^

**Table 5:** The inhibition of root elongation (calculated as a percentual inhibition of root growth compared to control) of white mustard (*Sinapis alba*), lettuce (*Lactuca sativa*) and clover (*Trifolium pratense*) after 3 days of seed germination in a solution supplemented with Zn_3_BO_6_ at concentrations of 10, 50, 100, 250, 500 and 1000 mg/L. Standard deviation is represented as ± S.D. (*n* = 3), and two-way ANOVA test with Dunnett’s multiple comparisons was applied.

	I [%]					
c [mg/L]	*S. alba*		*L. sativa*		*T. pratense*	
10	−86.2	± 8.34 ^b^	13.2	± 1.63 ^A^	−13.3	± 2.35 ^γ^
50	−107	± 4.17 ^a^	19.7	± 0.542 ^B^	10.3	± 3.34 ^α^
100	−119	± 2.37 ^a^	31.5	± 1.25 ^C^	3.65	± 7.22 ^α^
500	−105	± 7.78 ^a^	42.5	± 2.87 ^D^	35.7	± 3.24 ^β^
1000	−28.2	± 6.40 ^c^	53.2	± 3.13 ^E^	44.4	± 2.65 ^β^

## Data Availability

Data sharing is not applicable to this article.

## Supplementary Materials

The following supporting information can be downloaded at: https://www.mdpi.com/article/10.3390/toxics10120795/s1, Table S1: Steinberg medium; Table S2: Nutrient solution for acute toxicity test; Figure S1: Chlorophyll a/b ratio in *L. minor* plants.
