# Supplementary material for: Ecotoxicity Study of Additives Composed of Zinc and Boron
Source: Toxics. 2022 Dec 17;10(12):795. doi: 10.3390/toxics10120795 (PMC9782054; doi:10.3390/toxics10120795)
Supplement: Supplementary file 1 [file toxics-10-00795-s001.zip › Figure S1.pdf]

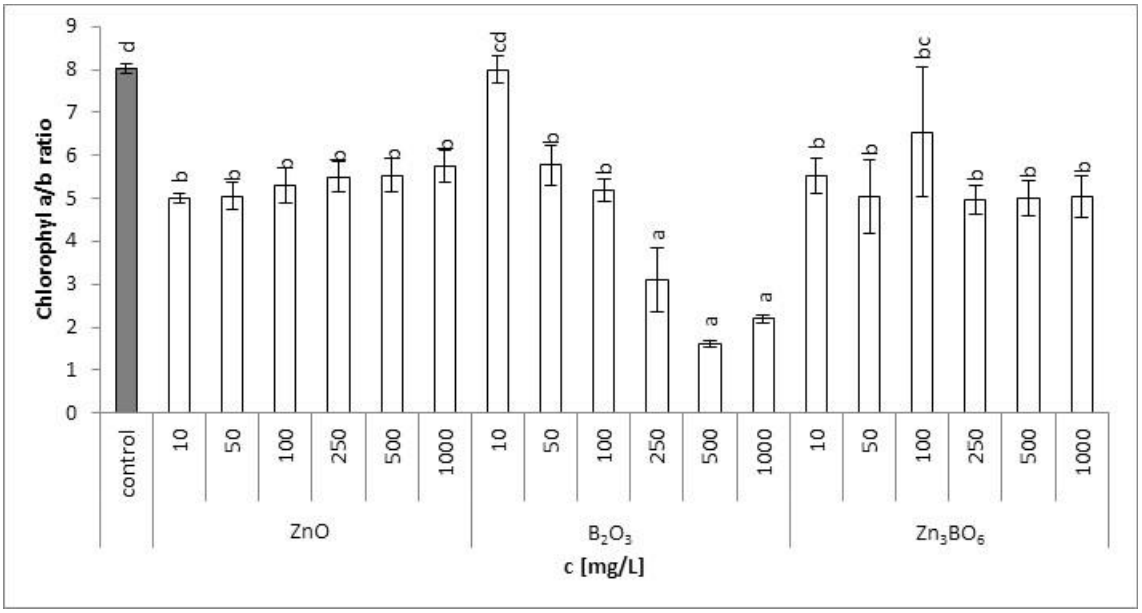

Figure S1. Chlorophyll a/b ratio in *L. minor* plants after 7 days of growth in the solutions supplemented with ZnO, B_2O_3, or Zn_3BO_6 at concentrations 10, 50, 100, 250, 500, and 1000 mg/L. Control plants grew in Steinberg solution; standard deviation is represented as ± S.D. (n = 3), and two-way ANOVA test with Dunnett's multiple comparisons was applied.
