# Supplementary material for: Ecotoxicity Study of Additives Composed of Zinc and Boron
Source: Toxics. 2022 Dec 17;10(12):795. doi: 10.3390/toxics10120795 (PMC9782054; doi:10.3390/toxics10120795)
Supplement: Supplementary file 1 [file toxics-10-00795-s001.zip › Table S1.pdf]

Table S1 Steinberg medium pH 5.5 ± 0.2 (adjusted by addition of a minimised volume of NaOH solution or HCl at ≤ 1 mol/L).

**Substance**		**Nutrient medium**	
Macroelements	MW [g/mol]	c [mg/L]	c [mmol/L]
KNO_3	101	350	3.46
$Ca(NO_3)_2 \cdot 4H_2O$	236	295	1.25
KH_2PO_4	136	90.0	0.66
K_2HPO_4	174	12.6	0.072
$MgSO_4 \cdot 7H_2O$	246	100	0.41
Microelements	MW [g/mol]	c [µg/L]	c [µmol/L]
H_3BO_3	61.8	120	1.94
$ZnSO_4 \cdot 7H_2O$	287	180	0.63
$Na_2MoO_4 \cdot 2H_2O$	242	44.0	0.18
$MnCl_2 \cdot 4H_2O$	198	180	0.91
$FeCl_3 \cdot 6H_2O$	270	760	2.81
EDTA Disodium- dihydrate	372	1 500	4.03
