# Supplementary material for: Ecotoxicity Study of Additives Composed of Zinc and Boron
Source: Toxics. 2022 Dec 17;10(12):795. doi: 10.3390/toxics10120795 (PMC9782054; doi:10.3390/toxics10120795)
Supplement: Supplementary file 1 [file toxics-10-00795-s001.zip › Table S2.pdf]

Table S2 Nutrient solution composition for acute toxicity test pH 7.8 ± 0.2 (adjusted by addition of a minimised volume of NaOH or HCl at < 1 mol/L).

Nutrient	c [mg/L]
$CaCl_2.2H_2O$	0.735
$MgSO_4.7H_2O$	0.275
$NaHCO_3$	0.162
KCl	0.0145
